# Immunoexpression of MMP-8 and MMP-9 in chronic subdural hematoma

**DOI:** 10.3389/fneur.2022.988854

**Published:** 2022-08-17

**Authors:** Gao-Jian Su, Di Zhang, Jia-Nuo Wu, Yu-Hang Deng, Chu-Wei Wu, Xie-Jun Zhang, Xian-Jian Huang

**Affiliations:** Department of Neurosurgery, Shenzhen Key Laboratory of Neurosurgery, The First Affiliated Hospital of Shenzhen University, Shenzhen Second People's Hospital, Shenzhen, China

**Keywords:** chronic subdural hematoma, matrix metallopeptidase, immunoexpression, differential gene expression, disease development

## Abstract

To determine the possible role of matrix metallopeptidase (MMP)-8 and MMP-9 in the development of chronic subdural hematoma (CSDH), we investigated their expression in CSDH. In our previous study, we analyzed hematoma fluid and peripheral blood of 83 patients with CSDH, including 17 postoperative patients. Based on these results, we included 50 people in the normal group and analyzed 20 markers in the peripheral blood of each person. In order to identify representative markers, it was assessed by using overall differential gene expression. The concentration of MMP-8 was significantly higher in the normal group than that in the preoperative and postoperative groups. The concentration of MMP-9 was significantly lower in the normal group than in both preoperative and postoperative groups. Immunohistochemistry confirmed the expression of MMP-8 and MMP-9 in CSDH membranes. In conclusion, our results provide evidence of the expression of MMP-8 and MMP-9 in CSDH. In addition, the expression of MMP-8 and MMP-9 suggests angiogenesis in CSDH formation.

## Introduction

As a common neurosurgical disease, chronic subdural hematoma (CSDH) involves the collection of blood in the subdural space (1). The incidence rates of CSDH have been rising on account of the aging population and the increasing use of anticoagulants and antiplatelet medications (2). However, the development of CSDH is still poorly understood (3, 4). Previous research has investigated the mechanisms underlying CSDH, including angiogenesis, fibrinolysis, and inflammation (5).

In a rat model, inflammation and angiogenesis were found to play important roles in the formation of CSDH (6, 7). Numerous biomarkers have been identified in patients with CSDH, including interleukin (IL)-1, IL-6, IL-8, IL-10, vascular endothelial growth factor (VEGF), matrix metallopeptidase (MMP)-2, and MMP-9 (5, 8–10). In our previous study, we found that MMP-8 and MMP-9 may play a key role in the pathophysiology of CSDH (11).

MMPs are a family of zinc-dependent proteolytic enzymes that contribute to pathological inflammation and endothelial dysfunction (12). This appears to be related to CSDH formation. However, there have been few studies on the expression and activity of MMPs in CSDH. Based on our previous study, we used immunohistochemistry to analyze CSDH membranes to determine the possible role of MMP-8 and MMP-9 in the development of CSDH (11).

## Materials and methods

### Patients and tissue samples

We recruited 50 individuals to the normal group based on our previous study (11). In addition, the membranes of CSDHs were obtained from 10 patients (eight males and two females) who underwent surgeries at the Shenzhen Second People's Hospital. The patients ranged from 35 to 84 years of age (mean: 65.3 years) and had not been previously treated for CSDH. This clinical study was approved by the Ethics Committee of the Shenzhen Second People's Hospital (20200422003-XZ2021-XZ2021). Informed consent was obtained from all the participants involved in this study.

### Cytokine measurements

Peripheral blood samples were collected from 50 patients in the control group. All samples were collected in tubes containing a coagulator and immediately centrifuged at 2,000 rpm for 15 min. After centrifugation, the supernatants were stored in sealed polypropylene tubes at −80 °C until further analysis.

We analyzed the hematoma fluid and preoperative and postoperative peripheral blood samples using the 20-plex human panel A system (R&D Systems, Minneapolis, MN, USA), Luminex system (Luminex, Austin, TX, USA), and Bioplex software (BioRad, Hercules, CA, USA). We evaluated IL-1α, IL-6, IL-8, IL-10, Angiopoietin-2, platelet-derived growth factor-BB (PDGF-BB), MMP-1, MMP-2, MMP-3, MMP-8, MMP-9, D-dimer, epidermal growth factor (EGF), C-C motif chemokine ligand 2 (CCL2), tumor necrosis factor (TNF-α), hepatocyte growth factor (HGF), VEGF, insulin-like growth factor binding protein-3 (IGFBP-3), prolactin, and VEGF receptor-2 (VEGFR2) levels.

### Differentially expressed gene screening

We searched the genes corresponding to cytokines through PubMed. The overall differential gene expression was evaluated by using the ggplot2 package in the R statistical software. Furthermore, the LIMMA package was used to select DEGs. We used the empirical Bayes moderated *t*-test to calculate the *p*-values for each cytokine. The adjusted *p*-values were calculated based on the false discovery rate. Only genes with a log_2_ fold change > 2 and false discovery rate < 0.01 were considered DEGs.

### Immunohistochemistry

CSDH membrane tissues were placed in 4% paraformaldehyde for 20 h at 4 °C. The tissue was embedded in paraffin, and 4 μm serial sections were cut. The sections were deparaffinized in xylene and dehydrated using descending dilutions of ethanol (100% and 95%). The primary antibodies (Goat monoclonal, Anti-MMP-8, No: AF908-SP, 1:50, R&D Systems; Rabbit monoclonal, Anti-MMP-9, No: ab76003, 1:50, Abcam, Cambridge, UK) were incubated for 30 min at 25 °C and overnight at 4 °C and stained using the avidin-biotin peroxidase method and 3, 3'-diaminobenzidine as a chromogen. Finally, the sections were counterstained with Mayer's hematoxylin and mounted using an aqueous mounting medium.

### Statistical analysis

Statistical analyses were performed using the Mann–Whitney *U*-test. Data are presented as mean ± standard deviation. All analyses were performed using the SPSS software. Statistical significance was set at *p* < 0.05.

## Results

### Patient characteristics

The mean age in the preoperative CSDH group was 67.16 ± 4.13 years. The general characteristics of each group are presented in Table 1.

**Table 1:** Age and sex in preoperative chronic subdural hematoma (CSDH), postoperative CSDH, and normal groups.

**Group**	**Age (years)**	**Gender**	
		**Male**	**Female**
Preoperative CSDH group	66.73 ± 15.14	76	7
Postoperative CSDH group	62.63 ± 15.17	17	0
Normal group	67.16 ± 4.13	34	16

### Identification of DEGs

All peripheral blood samples obtained from the preoperative CSDH and normal groups were analyzed. An overview of the differential gene expression is presented in Table 2. MMP-2, PDGF-BB, IL-10, EGF, IL-1α, CCL2, Angiopoietin-2, IL-6, MMP-8, VEGF, VEGFR2, HGF, MMP-9, IGFBP-3, TNF-α, IL-8, and Prolactin were DEGs between the preoperative and normal groups. MMP-1, MMP-3, and D-dimer were not significantly different between the preoperative and normal groups. All peripheral blood samples obtained from the postoperative and normal groups were analyzed. An overview of the differential gene expression is presented in Table 3. CCL2, EGF, IL-10, IL-1α, Angiopoietin-2, IL-6, MMP-8, HGF, TNF-α, MMP-9, IL-8, VEGFR2, VEGF, prolactin, D-dimer, IGFBP-3, MMP-1, and PDGF-BB were identified as DEGs between postoperative and normal groups. MMP-2 and MMP-3 were not significantly different between the postoperative and normal groups.

**Table 2:** Differential expression of all cytokines in peripheral blood samples between the preoperative CSDH and normal groups.

**Gene**	**logFC**	**AveExpr**	***t***	***p*** **-value**	**adj.p.Val**	***B***
MMP-2	921,853.2	647,819.4	32.36068	2.82 × 10^−63^	5.65 × 10^−62^	−3.4005
PDGF-BB	12,105.66	8,795.523	22.97331	4.09 × 10^−47^	4.09 × 10^−46^	−3.51766
IL-10	56.93396	46.3614	17.42905	1.61 × 10^−35^	1.07 × 10^−34^	−3.65504
EGF	572.5483	504.8891	15.49331	4.42 × 10^−31^	2.21 × 10^−30^	−3.72463
IL-1α	101.4245	62.9607	13.51954	2.25 × 10^−26^	8.99 × 10^−26^	−3.81145
CCL2	833.6593	744.0045	13.47686	2.85 × 10^−26^	9.51 × 10^−26^	−3.81352
Angiopoietin-2	3,864.163	3,543.209	12.12706	5.68 × 10^−23^	1.62 × 10^−22^	−3.88385
IL-6	59.11894	49.43651	10.86112	7.52 × 10^−20^	1.88 × 10^−19^	−3.95836
MMP-8	16,494.91	15,772.37	10.41476	9.49 × 10^−19^	2.11 × 10^−18^	−3.98664
VEGF	267.819	275.1433	9.980343	1.11 × 10^−17^	2.22 × 10^−17^	−4.01513
VEGFR2	10,538.92	15,761.35	9.8638	2.15 × 10^−17^	3.90 × 10^−17^	−4.02294
HGF	602.7063	669.2817	9.530186	1.41 × 10^−16^	2.34 × 10^−16^	−4.04566
MMP-9	−204,543	143,571	−9.10685	1.51 × 10^−15^	2.32 × 10^−15^	−4.07523
IGFBP-3	256,976.4	730,131.4	8.702437	1.42 × 10^−14^	2.03 × 10^−14^	−4.10423
TNF-α	70.08539	67.17581	7.703415	3.26 × 10^−12^	4.35 × 10^−12^	−4.17849
IL-8	76.15073	76.41651	5.749083	6.29 × 10^−08^	7.87 × 10^−08^	−4.32906
Prolactin	25,706.85	32,042.06	4.318917	3.13 × 10^−05^	3.68 × 10^−05^	−4.4343

*MMP, Matrix metallopeptidases; PDGF-BB, platelet-derived growth factor-BB; IL, interleukin; EGF, endothelial growth factor; CCL2, C–C motif chemokine ligand 2; VEGF, vascular endothelial growth factor; VEGFR2, VEGF receptor 2; HGF, hepat2ocyte growth factor; IGFBP-3, insulin-like growth factor binding protein-3; TNF-α, tumor necrosis factor-alpha; logFC, differential expression multiple; AveExpr, mean expression level; t, T value (paired-samples t-test); adj.p.Val, adjusted p-value; B, logarithm of the standard deviation after Bayes analysis*.

**Table 3:** Differential expression of all cytokines in peripheral blood samples between the postoperative CSDH and normal groups.

**Gene**	**logFC**	**AveExpr**	***t***	***p*** **-value**	**adj.p.Val**	***B***
CCL2	988.5303	1,032.101	21.16245	7.22 × 10^−30^	1.44 × 10^−28^	−4.03779
EGF	762.2283	682.9133	20.75541	2.06 × 10^−29^	2.06 × 10^−28^	−4.04051
IL-10	79.30262	63.22905	16.70569	1.71 × 10^−24^	1.14 × 10^−23^	−4.07664
IL-1α	118.1598	98.51254	15.5764	5.56 × 10^−23^	2.78 × 10^−22^	−4.09068
Angiopoietin-2	5,129.373	4,748.264	15.33773	1.19 × 10^−22^	4.74 × 10^−22^	−4.09395
IL-6	80.18971	67.46492	14.74762	7.92 × 10^−22^	2.64 × 10^−21^	−4.1025
MMP-8	23,775.32	20,468.85	14.63503	1.14 × 10^−21^	3.10 × 10^−21^	−4.10422
HGF	902.3919	832.87	14.61023	1.24 × 10^−21^	3.10 × 10^−21^	−4.1046
TNF-α	98.88158	87.63968	14.45219	2.08 × 10^−21^	4.63 × 10^−21^	−4.10706
MMP-9	−306,457	88,102.92	−14.0502	7.92 × 10^−21^	1.58 × 10^−20^	−4.11359
IL-8	113.0108	97.32476	10.75424	9.67 × 10^−16^	1.76 × 10^−15^	−4.18463
VEGFR2	13,529.04	19,157.61	10.10128	1.15 × 10^−14^	1.92 × 10^−14^	−4.20335
VEGF	355.4574	358.676	10.06004	1.35 × 10^−14^	2.07 × 10^−14^	−4.20459
Prolactin	40,781.43	38,473.71	3.968871	0.000192	0.000274	−4.47445
D-dimer	1,788,130	3,265,902	3.009334	0.003797	0.005062	−4.52305
IGFBP-3	135,979.4	859,112.6	2.618526	0.011113	0.013891	−4.54107
MMP-1	2,094.374	8,114.673	2.457844	0.01682	0.019789	−4.54806
PDGF-BB	2,048.92	15,908.91	2.352573	0.021871	0.024301	−4.55248

*MMP, Matrix metallopeptidases; PDGF-BB, platelet-derived growth factor-BB; IL, interleukin; EGF, endothelial growth factor; CCL2, C–C motif chemokine ligand 2; VEGF, vascular endothelial growth factor; VEGFR2, VEGF receptor 2; HGF, hepat2ocyte growth factor; IGFBP-3, insulin-like growth factor binding protein-3; TNF-α, tumor necrosis factor-alpha; logFC, differential expression multiple; AveExpr, mean expression level; t, T value (paired-samples t-test); adj.p.Val, adjusted p-value; B, logarithm of the standard deviation after Bayes analysis*.

### Data analysis

**Figure 1:**
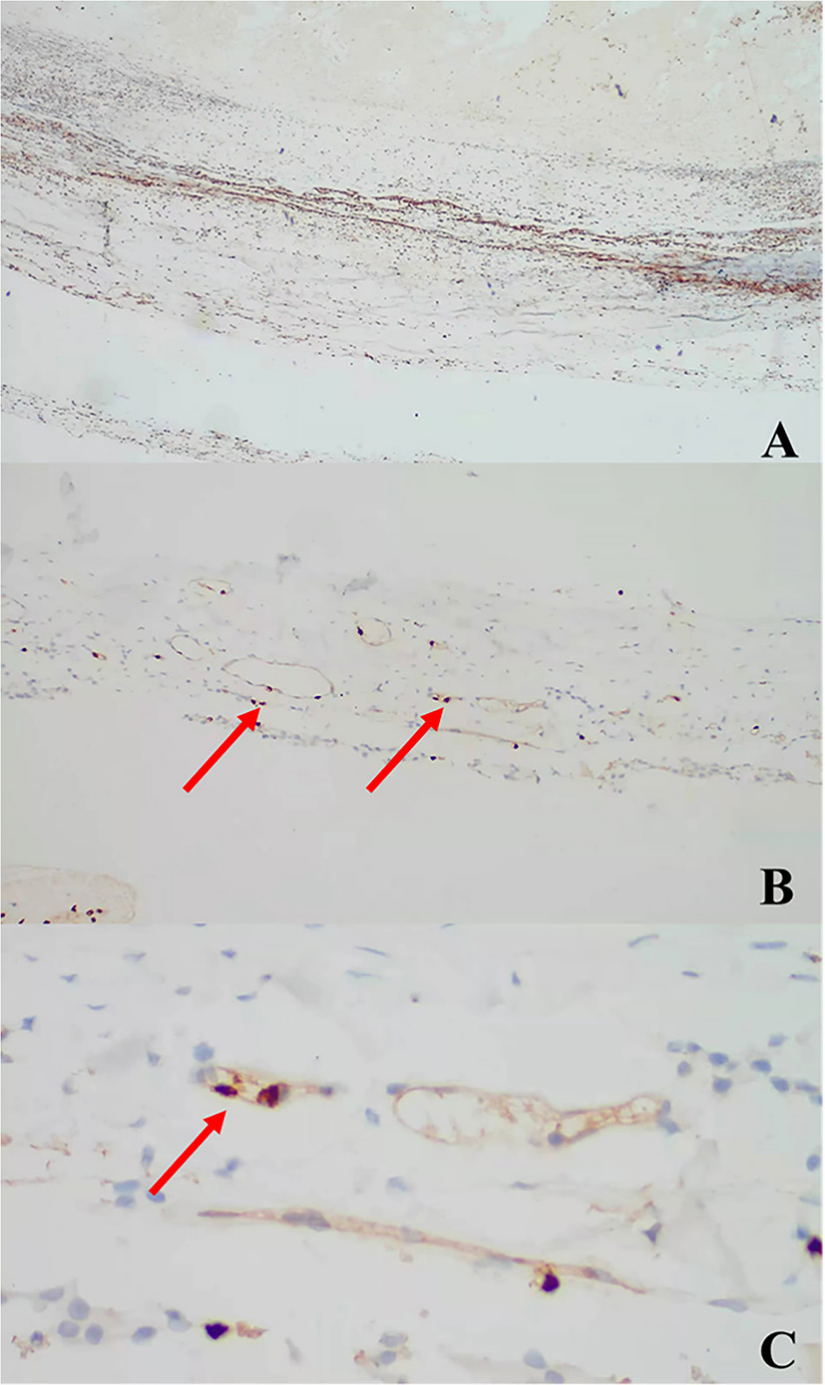
Anti-MMP-8 antibody immunostaining: **(A)** × 40, **(B)** × 100, **(C)** × 400 (arrows).

**Figure 2:**
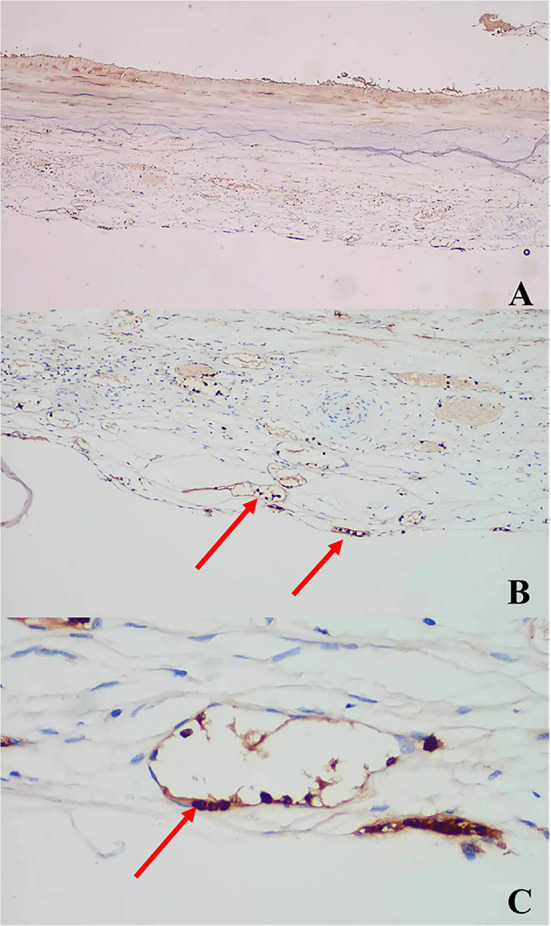
Anti-MMP-9 antibody immunostaining: **(A)** × 40, **(B)** × 100, **(C)** × 400 (arrows).

The concentrations of MMP-8 and MMP-9 in the control (normal) group are shown in Table 4 (p < 0.01). Based on our previous study (11), the concentration of MMP-8 was significantly higher in the normal group compared to that in both preoperative and postoperative CSDH groups, while that of MMP-9 was significantly lower. All CSDH membranes were immunostained for MMP-8 and MMP-9 in all samples. These proteins were positively immunostained in vascular endothelial cells. The results for the immunostained membranes are shown in Figures 1, 2.

**Table 4:** Concentration of MMP-8 and MMP-9 in the normal, preoperative, and postoperative groups (*p* < 0.01).

**Factor**	**MMP-8 (ng/mL)**	**MMP-9 (ng/mL)**
Preoperative serum	9.18 ± 9.78	217.19 ± 155.02
Normal serum	25.89 ± 6.09	15.178 ± 2.47
Postoperative serum	2.97 ± 3.80	354.13 ± 231.55

*MMP, Matrix metallopeptidases*.

## Discussion

Based on our previous study, MMP-8 and MMP-9 may contribute to CSDH pathogenesis in different ways (11). We found that the MMP-8 concentration was significantly higher in the normal group compared to that in preoperative and postoperative groups, whereas the MMP-9 concentration was significantly lower. Simultaneously, we observed that both MMP-8 and MMP-9 were expressed in vascular endothelial cells. Nowadays, an increasing number of studies consider that CSDH formation may be related to the growth of new vessels and inflammation (1, 13). In addition, MMPs significantly contribute to blood vessel formation, remodeling, and angiogenesis by regulating the functions or behaviors of stem/progenitor and vascular cells (14).

Although CSDH is the most common neurosurgical disease, a few questions remain to be answered. Tamura et al. (15) indicated that the split dural border cell layer produced a dural hematoma by forming the inner and outer membranes in CSDH. The outer membranes drive inflammation and angiogenesis. After studying the role of MMPs in the development of CSDH, Nakagawa et al. (16) found that MMPs degrade the integrity of the extracellular matrix in the outer membranes of CSDH, which can cause the exudation of interstitial edematous fluid from membrane vessels into the hematoma cavity. This seemed to be the reason for CSDH enlargement. Some studies have shown that MMP-8 plays an important role in endothelial cell angiogenesis (12, 14). In our study, the change in the MMP-8 concentration indicated that MMP-8 might be inhibited when neovascularization proceeds and increases after the formation of new vessels are suspended. In future studies, we will focus on MMP-8 in CSDH formation.

The role of MMP-9 in the pathophysiological pathways of CSDH has been reported in many studies (1, 10, 11, 13). MMP-9 expression in vascular endothelial cells confirmed that CSDH formation was related to MMP-9 expression. MMP-9 is regarded as one of the factors contributing to the formation of fragile, leaky capillaries (5). Hua et al. (10) considered that the MMP/VEGF system may be involved in angiogenesis associated with CSDH, because of the relation of the concentrations of MMP-2 and MMP-9 and the VEGF concentration in the hematoma fluid. Xu et al. (6) found that the formation of neovessels might be correlated with the increased production of pro-angiogenic factors, including MMP-9 and VEGF, in rats. Furthermore, studies have indicated that MMP-9 can reduce CSDH absorption by increasing vascular permeability, enhancing inflammation, and reducing vascular maturation (13). Huang et al. (3) found that atorvastatin plays a similar role to MMP-9 and can promote hematoma absorption, reduce tissue inflammation, and improve neurological function. We propose that when the trigger began, the MMP-9 concentration increased rapidly, while that of MMP-8 decreased. These biomolecules interact with and may influence each other. With the development and treatment of CSDH, the concentration of MMP-9 also increased, indicating that it could promote the formation of new normal vessels to repair normal physiological function.

Our understanding of CSDH pathogenesis is constantly being updated, and researchers are currently focusing on developing appropriate treatments. It has been found that atorvastatin inhibits inflammation and angiogenesis (17, 18). Based on a study of SDH in rats, it may eliminate SDH and improve neural function by using atorvastatin (19). In addition, Soleman et al. (20) concluded that atorvastatin is valid and safe for treating asymptomatic or mildly symptomatic patients with CSDH. Atorvastatin may enhance angiogenesis to reduce CSDH-related inflammation (21). Fan et al. (22) found that Krüppel-like factor 2 (KLF-2) plays a key role in drug therapy for CSDH. It was found that combination therapy with atorvastatin and low-dose dexamethasone can inhibit the expression of KLF-2 to attenuate robust endothelial inflammation and permeability (22). In a randomized placebo-controlled trial, effective function was confirmed in patients treated with atorvastatin and low-dose dexamethasone (23). Our findings indicate that MMP-8 and MMP-9 may serve as accessible markers of CSDH formation. Future studies should focus on the relationship between MMP-8 and MMP-9 in CSDH fluid and their membrane activity. Moreover, we wish to determine the relationship between these two cytokines and CSDH prognosis and identify a way to prevent recurrence in patients with CSDH. More important in the clinic, understanding CSDH mechanisms would help physicians restore neurological deficits in patients with CSDH and improve their quality of life (24).

There were some limitations in this study. Firstly, from our limited number of cases, it is difficult to represent the wide spectrum of patients with CSDH. Further studies including a larger sample of patients will be needed to clarify this point. Secondly, the experimental analysis of the CSDH membrane was relatively limited, and so the results may not be completely convincing. More approaches are needed to verify the expression and role of MMP-8 and MMP-9 on the membrane.

## Conclusions

Our study provides evidence of MMP-8 and MMP-9 expression in CSDH. In addition, the expression of MMP-8 and MMP-9 is indicative of angiogenesis in CSDH formation. Identification of the biomolecules involved in CSDH formation may be helpful in the development of new clinical therapies. However, further studies are necessary to clarify the association between MMP-8 and MMP-9 expression during the transformation and progression of CSDH.

## Data availability statement

The original contributions presented in the study are included in the article/supplementary material, further inquiries can be directed to the corresponding author.

## Ethics statement

The studies involving human participants were reviewed and approved by Institutional Ethics Committee of Shenzhen Second People's Hospital. Written informed consent to participate in this study was provided by the participants' legal guardian/next of kin.

## Author contributions

Conceptualization, methodology, and project administration: G-JS, DZ, and X-JH. Validation, resources, and investigation: G-JS and DZ. Software: J-NW, C-WW, and X-JZ. Formal analysis: Y-HD, C-WW, and X-JZ. Data curation, visualization, and writing—original draft preparation: G-JS. Writing—review and editing and supervision: X-JH. Funding acquisition: X-JH and X-JZ. All authors contributed to the article and approved the submitted version.

## Funding

This research was funded by the Natural Science Foundation of China, Grant Number 81301062, Shenzhen Double Chain Grant, Grant Number (2018) 256, Discipline Construction Capacity Improvement Project of the Shenzhen Municipal Health Commission, Grant Number SZXJ2018057, and Basic Research Fund of the Shenzhen Science and Technology Program, Grant Numbers JCYJ20180228163034627 and JCYJ20190806162210754.

## Conflict of interest

The authors declare that the research was conducted in the absence of any commercial or financial relationships that could be construed as a potential conflict of interest.

## Publisher's note

All claims expressed in this article are solely those of the authors and do not necessarily represent those of their affiliated organizations, or those of the publisher, the editors and the reviewers. Any product that may be evaluated in this article, or claim that may be made by its manufacturer, is not guaranteed or endorsed by the publisher.
